# Is the cochlear implant a successful long-term solution for single-sided deaf and asymmetric hearing-impaired patients?

**DOI:** 10.1007/s00405-020-06411-y

**Published:** 2020-10-16

**Authors:** Iva Speck, Pascal Challier, Thomas Wesarg, Till Fabian Jakob, Antje Aschendorff, Frederike Hassepass, Susan Arndt

**Affiliations:** Faculty of Medicine, Department of Otorhinolaryngology–Head and Neck Surgery, Medical Center–University of Freiburg, Killianstraße 5, 79106 Freiburg, Germany

**Keywords:** Single-sided deafness, Asymmetric hearing loss, Cochlear Implant, Long term

## Abstract

**Purpose:**

We investigated the long-term results of cochlear implant (CI) recipients with asymmetric hearing loss (AHL) or single-sided deafness (SSD). We focused on wearing behavior, audiometric hearing rehabilitation, and subjective benefits of the CI. CI is expected to improve audiological results, subjective hearing perception, and tinnitus burden.

**Methods:**

Speech recognition in background noise and sound localization were assessed preoperatively and after at least six years of CI experience. Validated questionnaires determined the subjective benefit of CI use and the subjective evaluation of tinnitus.

**Results:**

Over 80% of the included AHL and SSD CI recipients used their CI between 6 and 10 h daily; four subjects with SSD were non-users. Speech recognition in background noise and sound localization improved significantly compared with the unaided preoperative situation. Additionally, CI improved subjective speech intelligibility and spatial hearing impression while reducing tinnitus burden.

**Conclusion:**

Subjects with AHL and SSD benefit from CI, subjectively and audiologically. Cochlear implant is a successful long-term treatment for AHL and SSD.

## Introduction

The extreme asymmetry of hearing impairs individuals with single-sided deafness (SSD) and asymmetric hearing loss (AHL) in their speech recognition in noise and the localization of sound sources [1–5]. SSD and AHL can also cause psychological health issues, e.g., high-stress levels, high self-reported listening effort, low self-efficiency, and low self-confidence, resulting in exhaustion, frustration, and social withdrawal [5–8]. In addition, many AHL and SSD patients experience disturbing tinnitus [2, 9–11].

Cochlear implants (CIs) improve speech recognition in noise and the localization of sound sources in patients with SSD [12–15]. CIs also reduce the cognitive load and improves the anxiety states and hearing-related quality of life of such patients [1, 2, 10, 11, 16].

In the first and, to date, only long-term study on SSD CI recipients, Távora-Vieira and colleagues compared the conditions of CI turned on and CI turned off at the time of long-term measurement (follow-up of 4–10 years) [14].

Our aim was to determine the long-term success of CI in subjects with SSD and AHL by comparing preoperative with long-term measurements. To investigate successful treatment, we collected the following data: the wearing behavior of the CI, speech recognition in noise, the localization of sound sources, the subjective evaluation of hearing, and the tinnitus burden. We expected continuous use in cases of treatment success and improvement of the audiological results, subjective hearing and tinnitus burden.

## Materials and methods

The Ethics Committee of the Albert-Ludwigs-University of Freiburg (No. 3/17) approved the present study. We registered the study with the German Register of Clinical Studies (DRKS00017632).

### Participants

We derived the criteria for SSD and AHL from the consensus papers of Vincent et al. [17] and Van de Heyning et al. [18]. These consensus papers require the poorer-hearing ear to have an unaided hearing threshold of  ≥ 70 dB HL up to and including 4 kHz. For the better-hearing ear, we applied the definition by Vincent and colleagues [17], namely SSD participants: hearing threshold of  ≤ 30 dB HL in the frequencies up to and including 4 kHz; AHL participants: hearing impairment of  > 30 dB HL and  ≤ 60 dB HL up to and including 4 kHz [17]. The interaural asymmetry was required to be equal to or more than 30 dB [17].

We included German-speaking adults treated at the ENT University Hospital Freiburg between 2008 and 2013. "Long-term" was defined as a period of five or more years after the initial fitting of the CI and, in the present study, ranged from six to eleven years.

### Data collection

We compared long-term measurements with preoperative measurements. We chose the measurements that Vincent et al. and Van de Heyning et al. proposed as evidence of the therapeutic success of CI in subjects with SSD and AHL [17, 18].

### Audiometric measurement

We performed bone- and air-conduction pure-tone audiometry and the unaided Freiburg monosyllable test in % at 65 dB SPL. Audiometric data were compared using the pure-tone average for the frequencies 500, 1000, 2000, and 4000 Hz (pure-tone average, PTA4).

Speech recognition in background noise was tested with the Oldenburg sentence test (OLSA) [19, 20]. The 50% speech intelligibility threshold was determined adaptively with a fixed noise level of 65 dB SPL and an initial speech level of 65 dB SPL. We examined three presentation conditions: S0N0, SnhNssd, and SssdNnh, as previously described by Arndt and colleagues [2]. In the presentation condition S0N0, both speech and noise were displayed from the front at an angle of 0°. In the presentation condition SnhNssd, the speech was presented from the better-hearing side and background noise from the poorer-hearing side at an angle of 45°. Speech and noise presentation was vice versa in the presentation condition SssdNnh: speech from the poorer-hearing side and background noise from the better-hearing side, each at an angle of 45°.

We used an array of 7 speakers at head level in a frontal semicircle to assess localization, as previously described by Arndt and colleagues [1, 2]. The loudspeakers were set up in a range of  – 90° to 90° with a separation angle of 30°. Each localization test consisted of 70 OLSA sentences presented randomly from one loudspeaker at sound levels of 59, 62, 65, 68, and 71 dB SPL (mean sound level of 65 dB SPL). For each participant and each condition, the localization ability was measured as the angle error in degrees, i.e., the mean angle distance between the presentation loudspeaker and the loudspeaker identified. The angle error corresponding to the chance level performance of correct identification was 68.6°.

### Subjective assessment

We measured the subjective outcome of CI with the German version of the standardized "Speech, Spatial and Qualities of Hearing Scale" (SSQ, version 3.1.2) [21]. SSQ evaluates speech intelligibility, spatial hearing, and quality of hearing in three sub-sections with 50 questions each (values between 0 and 10); the higher the score, the better the subjective assessment.

We evaluated the tinnitus burden with a numerical rating scale (NRS) between 0 and 10; 10 representing the highest tinnitus burden [22].

### Statistical analysis

We performed a statistical analysis in R (R Core Team 2017). We analyzed the SSD and AHL CI recipients separately. Significance was defined at a level of 0.05.

We examined the normal distribution with the Shapiro–Wilk test. Paired *t* tests (normal distribution) or Wilcoxon ‘s Ranks tests (not normal distribution) were used to compare the audiometric and subjective results between preoperative and long-term measurements (Table 1).

**Table 1:** Normal distribution and statistical test

Parameter	Normal distribution^1^	Statistical test^2^
Bone-conducted PTA4		
SSD	Non-parametric	Paired Wilcoxon-Ranks tests
AHL	Non-parametric	Paired Wilcoxon-Ranks tests**
Air-conducted PTA4		
SSD	Non-parametric	Paired Wilcoxon-Ranks tests
AHL	Non-parametric	Paired Wilcoxon-Ranks tests**
Monosyllabically speech recognition (unaided, 65 dB SPL)		
SSD	Non-parametric	Paired Wilcoxon-Ranks tests
AHL	Non-parametric	Paired Wilcoxon-Ranks tests*
Speech recognition in noise SssdNnh		
SSD	Parametric	Paired *t*-test***
AHL	Parametric	Paired *t*-test***
Speech recognition in noise – S0N0		
SSD	Parametric	Paired *t*-test
AHL	Parametric	Paired *t*-test
Speech recognition in noise – SnhNssd		
SSD	Parametric	Paired *t*-test
AHL	Parametric	Paired *t*-test
Localization of sound sources		
SSD	Parametric	Paired *t*-test***
AHL	Non-parametric	Paired Wilcoxon-Ranks tests***
SSQ – Speech intelligibility		
SSD	Parametric	Paired *t*-test***
AHL	Parametric	Paired *t*-test*
SSQ – Spatial hearing		
SSD	Parametric	Paired *t*-test***
AHL	Parametric	Paired *t*-test**
SSQ – Quality of auditory impression		
SSD	Parametric	Paired *t*-test
AHL	Parametric	Paired *t*-test
Tinnitus burden		
SSD	Non-parametric	Paired Wilcoxon-Ranks tests**
AHL	Non-parametric	Paired Wilcoxon-Ranks tests***

*AHL *asymmetric hearing loss, *SSD *single-sided deafness, *PTA4 *pure-tone average of the frequencies 500 Hz, 1 kHz, 2 kHz, and 4 kHz, *SssdNnh *speech from the poorer-hearing/implanted side and noise from the better-hearing side; at an angle of 45° and  – 45° each, *S0N0 *speech and noise from the front; at an angle of 0°, *SnhNssd *speech from the better-hearing side and noise from the poorer-hearing / implanted side; at angles of 45° and  – 45° each, *SSQ *Speech, Spatial, and Qualities of Hearing Scale

^1^Shapiro-Wilk test; ^2^comparison between preoperative and long-term measurement; *p < 0.05; **p < 0.01; ***p < 0.001

## Results

### Participation and wearing behavior

**Fig. 1:**
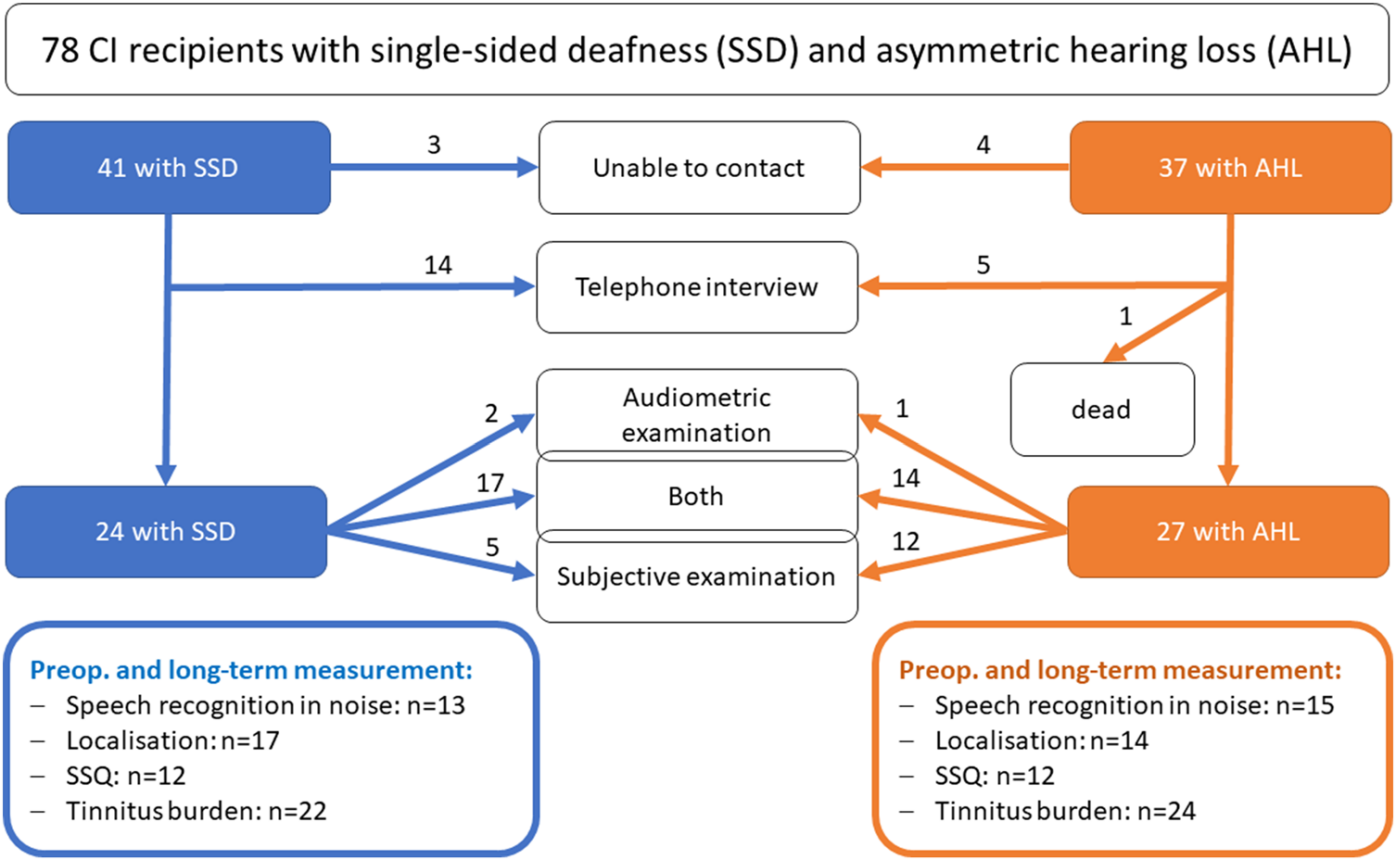
Flow chart of the participation of the CI recipients with single-sided deafness (SSD; blue) and asymmetric hearing loss (AHL; orange)

We identified 78 CI recipient with SSD or AHL and CI experience of between six and eleven years: 41 with SSD and 37 with AHL. Figure 1 displays the participation of each identified candidate. The CI recipients questioned by telephone reported only their wearing behavior and, if applicable, causes of non-use.

Most interviewed subjects, namely 34 SSD CI recipients (34/38) and 32 AHL CI recipients (32/32), used their speech processor between 6 and 10 h daily (median: 8 h) at six to eleven years after the initial fitting.

Four SSD CI recipients reported that they no longer wore their CI (9.8% non-users). All non-users agreed to a telephone interview but refused further testing. The non-users reported the following causes for non-use: (1) no speech comprehension with CI, (2) fear of contamination of CI in the workplace, (3) lack of practice with the CI, and (4) lack of subjective benefit. The non-user reporting the lack of speech comprehension was implanted with the CI one year after labyrinthitis [23]. The implantation was delayed because of the lack of coverage by the health insurance company. No AHL CI recipients reported as being non-users.

Table 2 displays the descriptive characteristics of the 24 SSD and 27 AHL CI recipients participating in either or both the audiometric and subjective examinations.

**Table 2:** Descriptive characteristics of the study groups, mean ± SD or *N* (%)

Characteristics	SSD CI recipients	AHL CI recipients
*N*	24	27
Femal	15 (63%)	15 (56%)
Age (years)	50.9 ± 10.9	63.8 ± 9.5
Duration of hearing impairment (years)	3.5 ± 7.4	7.8 ± 1.3
CI experience (years)	8.7 ± 1.9	4.3 ± 3.7
Cause of deafness		
Sudden deafness	10	9
Labyrinthitis	4	2
Endolymphatic hydrops	2	1
Mumps infection	1	1
Otosclerosis	2	1
After ear surgery	2	2
Petrous bone fracture	1	0
Cogan syndrome	1	0
Measles infection	1	1
After acoustic neuroma resection	0	1
After cerebral apoplexy	0	1
Unknown	0	8
CI manufacturer		
Cochlear	21	21
Med-El	3	5
Advanced Bionics	0	1

*AHL *asymmetric hearing loss, *CI *cochlear implant, *SSD *single-sided deafness

### Audiometric measurement

#### SSD CI recipients

For the better-hearing ear, we found no difference between preoperative and long-term measurement in bone-conducted PTA4 (6.1 ± 4.0 dB SPL to 6.6 ± 7.5 dB SPL), air-conducted PTA4 (10.9 ± 4.9 dB SPL to 12.9 ± 8.7 dB SPL), and unaided monosyllabically speech recognition at 65 dB SPL.

**Fig. 2:**
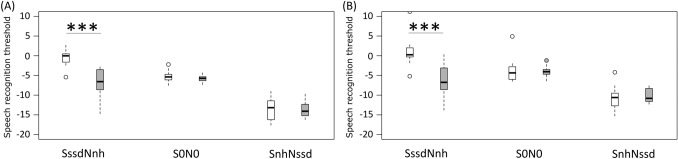
Box-whisker plots of the 50% speech recognition threshold against noise preoperatively (white) and long-term measurement after six to eleven years of CI experience (gray). SssdNnh = speech from the poorer-hearing/implanted side and noise from the better-hearing side; at an angle of 45°; S0N0 = speech and noise from the front; at an angle of 0°; SnhNssd = speech from the better-hearing side and noise from the poorer-hearing / implanted side; at angles of 45°. **a** 18 SSD CI recipients and **b** 15 AHL CI recipients

In the presentation condition SssdNnh, speech recognition improved significantly from an average of  –0 .6 ± 1.9 dB SPL in the preoperative unaided measurement to an average of  – 6.9 ± 3.2 dB SPL in the long-term measurement with CI (Fig. 2a and Table 1). The presentation conditions S0N0 and SnhNssd did not differ significantly (Fig. 2a).

**Fig. 3:**
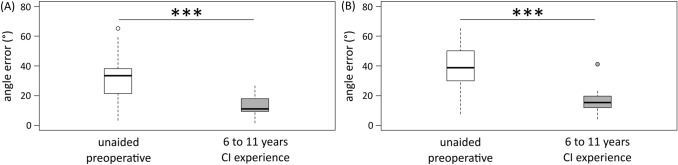
Box-whisker plots of the localization of sound sources preoperatively (white) and the long-term measurement after six to eleven years of CI experience (gray). **a** 17 SSD CI recipients and **b** 14 AHL CI recipients

The localization ability improved from an angle error from 33.4° to 11.3° (Fig. 3a and Table 1).

#### AHL CI recipients

The hearing ability in the better-hearing ear decreased significantly in bone-conduction PTA4 (21.1 ± 8.5 dB SPL to 25.6 ± 10.7 dB SPL), air-conduction PTA4 (26.6 ± 7.8 dB SPL to 31.9 ± 12.1 dB SPL), and unaided monosyllabic speech recognition at 65 dB SPL (Table 1).

In the presentation condition SssdNnh, speech recognition improved significantly from an average of 0.9 ± 3.6 dB SPL in the preoperative measurement to an average of 6.0 ± 4.0 dB SPL in the long-term measurement (Fig. 2b and Table 1). Speech recognition did not change significantly in the presentation conditions S0N0 and SnhNssd (Fig. 2b).

The ability to localize sounds improved significantly from an angle error of 38.1–16.7° (Fig. 3b and Table 1).

### Subjective assessment

#### SSD CI recipients

**Fig. 4:**
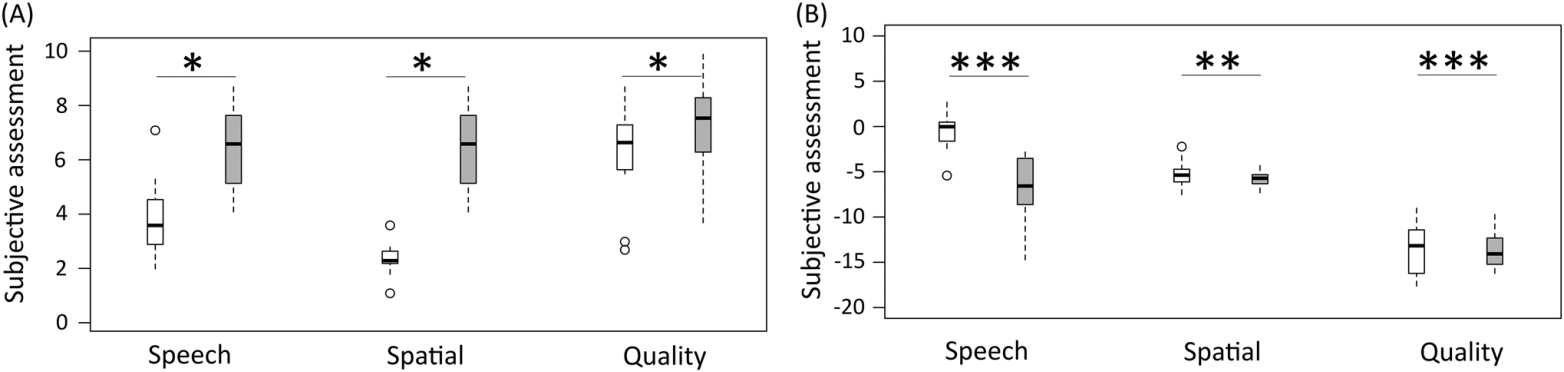
Box-whisker plots of the subjective assessment on the “Speech, Spatial, and Qualities of Hearing Scale” (SSQ) preoperatively (white) and long-term measurement after six to eleven years of CI experience (gray). **a** 12 SSD CI recipients and **b** 12 AHL CI recipients

**Fig. 5:**
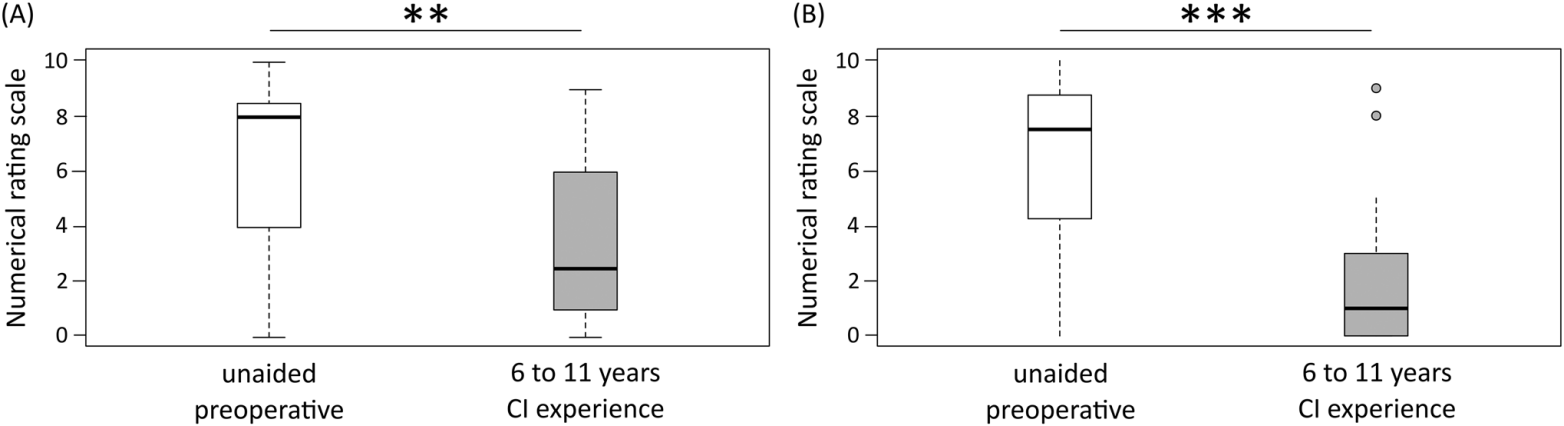
Box-whisker plots of the burden of tinnitus preoperatively (white) and long-term measurement after six to eleven years of CI experience (gray). **a** 22 SSD CI recipients and **b** 24 AHL CI recipients

Subjective speech intelligibility and spatial hearing in the SSQ improved significantly in the long-term measurement (Fig. 4a and Table 1). The quality of the auditory signal did not reach significance (*p* = 0.059). Of the 22 SSD CI recipients, 21 reported a preoperative tinnitus. The CI reduced the tinnitus burden significantly from 6.5 ± 2.8 to 3.4 ± 2.9 (Fig. 5a and Table 1).

#### AHL CI recipients

Subjective speech comprehension and spatial hearing improved significantly in the long-term measurement (Fig. 4b and Table 1). Of 24 AHL CI recipients, 21 reported tinnitus preoperatively. We found that CI significantly reduced the tinnitus burden in AHL CI recipients from 6.2 ± 2.1 to 3.5 ± 2.8 (Fig. 5b and Table 1).

## Discussion

We have found that CI improves speech recognition in noise, the localization of sound sources, and subjective speech intelligibility and spatial hearing in AHL and SSC CI recipients with six to eleven years of CI experience. CI also reduces the tinnitus burden in both groups. Other than four non-users with SSD, all interviewed patients wore their CI daily.

### Wearing behavior

Information on wearing behavior was available from 38 (92.7%) of the 41 long-term SSD CI recipients and from 32 (89.2%) of the 37 AHL CI recipients. Most of the interviewed SSD (89.5%) and all AHL (100%) CI recipients wore their CI for six to ten hours daily. The percentage of non-users was comparable with that for bilateral deaf patients at a rate of 2.8–13% [24, 25]. None of the four non-users terminated CI use because of equipment failure or perioperative complications. Three non-users reported that insufficient practice time or special workplace situations led to incomplete hearing rehabilitation and the lack of subjective benefit. We, therefore, recommend intensive preoperative education and consideration of each patient’s history before cochlea implantation to prevent non-use.

### Audiometric measurement

The CI enables a subject with AHL or SSD to overcome the head shadow when speech is presented on the side of the CI (SssdNnh) and thus ensures responsiveness on the CI-supplied side, even against noise. In this long-term study, we have confirmed the improvement in speech recognition against background noise in the condition SssdNnh as shown previously for shorter follow-up periods [1, 2, 12, 15, 26, 27].

When presenting speech and noise from the front (S0N0), we determined no change in speech recognition with CI, as earlier reported by our research group after a shorter CI follow-up [1, 2]. However, other authors have described significant improvement in this presentation condition, even in short-term evaluations [3, 14, 15, 27]; Távora-Vieira and colleagues (2019) have also reported this effect in their long-term study. Individual differences of the patients might cause this discrepancy: subjects included in the present study also participated in the shorter follow-up studies of our research group [1, 2]. Another contributing factor might be the conditions that were compared: Távora-Vieira and colleagues [14] compared the conditions CI “on” and “off”, whereas we compared preoperative with long-term results.

Speech recognition remained stable, even when the speech was presented from the NH side and noise came from the CI side (SnhNssd). In this presentation condition, we expected no improvement because of the asymmetric hearing situation and former study results [1, 2, 12, 14, 15, 26].

CI improved sound localization compared with the preoperative unaided situation. This agrees with the literature describing patients with shorter [1, 2, 26–29] and with longer CI experience [14].

### Subjective assessment

Speech comprehension and spatial hearing in the SSQ improved with six to eleven years of CI experience. Rating of long-term hearing quality was unchanged, similar to patients with six to twelve months of CI experience [1, 2]. However, in SSD CI recipients, we saw a trend towards better long-term hearing quality, despite the different acoustic and “electrical” hearing impressions; this did not reach statistical significance. Távora-Vieira and colleagues [14, 15] demonstrated a subjective improvement in the overall score of the SSQ; the SSQ subcategories were not evaluated separately.

CI permanently reduced tinnitus for most AHL (18/21) and SSD CI recipients (15/21). In 2 AHL and five SSD CI recipients, the intensity of the tinnitus remained unchanged, and only in one AHL and one SSD CI recipient was the tinnitus burden increased after CI surgery.

### Strength and limitations

The presented study has the longest follow-up period (6–11 years) of SSD CI recipients in the literature, followed by Távora-Vieira et al. (4–10 years) [14]. We are the first to compare preoperative to long-term measurements. We have been able to gain information about the wearing behavior of 92.7% of the SSD and 86.5% of the AHL CI recipients (Fig. 1).

From a total of 78 subjects, only 24 SSD and 27 AHL CI recipients participated in the audiological and subjective measurements. All known non-users only agreed to the telephone interview. Thus, perhaps, only the better performers agreed to audiological and subjective assessments; this bias cannot be ruled out with certainty. Because of incomplete follow-up data, e.g., 12 months after CI fitting, we were unable to include these measurements in the present study. We report wearing behavior in only two categories: “user” and “non-user”. Datalogging provides more detailed information on daily device use and environments; these data were not available for most participants. In a previous study, we examined the datalogging in patients using the Nucleus 6 device. Across age groups, SSD subjects had an active CI-time of 8.07 h/day; the age group 18 to 65 years used their CI for 7.73 h/day and subjects over the age of 65 years used their CI for 10.71 h/day [30]. In the present study, we focused on measurements recommended by the consensus papers on the successful treatment of SSD patients [17, 18].

### Implications for future research

Future studies should measure speech recognition in everyday-life relevant acoustic environments with interfering sound, e.g., in a restaurant or a busy street environment, to enhance applicability. Similarly, the localization set-up could be altered to mimic everyday life more closely. The inclusion and comparison of additional time points, e.g., 6 months and 12 months after initial fitting, would allow for the investigation of time-dependent changes in SSD and AHL CI recipients. Including further questionnaires on tinnitus, vertigo, and hearing-related quality of life might provide additional information.

## Conclusion

The cochlear implant is a successful long-term treatment for subjects with SSD and AHL. Only a small number of subjects with SSD are non-users after more than 5 years. Most of them stopped using the CI within the first year after implantation. Subjects with AHL and SSD can use their CI to overcome the head shadow effect without being disturbed by noise on the CI side, even in the presence of background noise. Cochlear implants improve subjective speech intelligibility and spatial hearing while reducing the tinnitus burden.

## Abbreviations

CI: Cochlear implant
NH: Normal hearing
AHL: Asymmetric hearing loss
SSD: Single-sided deafness
PTA4: Pure-tone average for the frequencies 500, 1000, 2000, and 4000 Hz
OLSA: Oldenburg sentence test
SSQ: Speech, Spatial and Qualities of Hearing Scale
NRS: Numerical rating scale
